# Thermodynamic Origin of the Photostability of the Two-Dimensional Perovskite PEA_2_Pb(I_1–*x*_Br_*x*_)_4_

**DOI:** 10.1021/acsenergylett.2c02463

**Published:** 2023-01-13

**Authors:** Zehua Chen, Haibo Xue, Geert Brocks, Peter A. Bobbert, Shuxia Tao

**Affiliations:** † Materials Simulation and Modelling, Department of Applied Physics, Eindhoven University of Technology, 5600 MBEindhoven, The Netherlands; ‡ Center for Computational Energy Research, Department of Applied Physics, Eindhoven University of Technology, P.O. Box 513, 5600 MBEindhoven, The Netherlands; ¶ Computational Materials Science, Faculty of Science and Technology and MESA+ Institute for Nanotechnology, University of Twente, P.O. Box 217, 7500 AEEnschede, The Netherlands; § Molecular Materials and Nanosystems, Eindhoven University of Technology, P.O. Box 513, NL-5600 MBEindhoven, The Netherlands

## Abstract

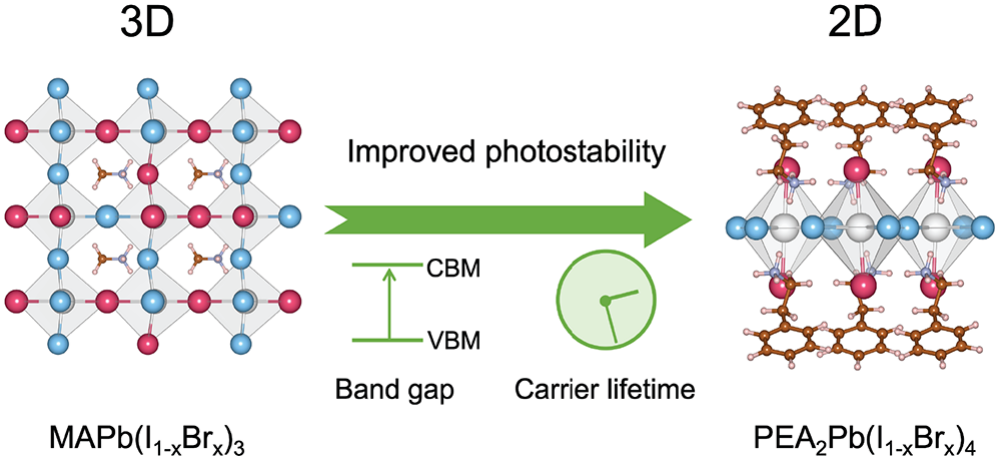

The two-dimensional (2D) mixed halide perovskite PEA_2_Pb(I_1–*x*_Br_*x*_)_4_ exhibits high phase stability under illumination as compared to the three-dimensional (3D) counterpart MAPb(I_1–*x*_Br_*x*_)_3_. We explain this difference using a thermodynamic theory that considers the sum of a compositional and a photocarrier free energy. Ab initio calculations show that the improved compositional phase stability of the 2D perovskite is caused by a preferred I–Br distribution, leading to a much lower critical temperature for halide segregation in the dark than for the 3D perovskite. Moreover, a smaller increase of the band gap with Br concentration *x* and a markedly shorter photocarrier lifetime in the 2D perovskite reduce the driving force for phase segregation under illumination, enhancing the photostability.

Metal-halide perovskites are rapidly emerging as a new class of semiconducting materials for photovoltaics. They have the general chemical formula ABX_3_, where A is a monovalent organic or inorganic cation like methylammonium (MA), formamidinium (FA), or Cs, M is a divalent metal cation like Pb or Sn, and X is a halide anion like I, Br, or Cl.^1−6^ By sandwiching three-dimensional (3D) ABX_3_ perovskite layers with large organic spacer cations, layered two-dimensional (2D) perovskites can be obtained.^7−11^ Among these, Ruddlesden–Popper (RP) perovskites have been widely studied as light absorbers owing to their superior moisture resistance.^12−16^ The general chemical formula of RP-type halide perovskites is R_2_A_*n*–1_B_*n*_X_3*n*+1_, where R is a monovalent organic spacer cation, e.g., phenethylammonium (PEA) or butylammonium (BA),^9,10^ and *n* is the number of B–X octahedral layers. Recently, the band gap tunability of 2D RP halide perovskites by compositional alloying on X sites has attracted increasing attention,^17−21^ which stimulated the use of these perovskites in solar cells, light-emitting devices, and photodetectors.^8,22−24^ Very promising is the recent finding that light-induced halide segregation, which occurs in the 3D perovksite MAPb(I_1–*x*_Br_*x*_)_3_ perovskite when the Br concentration *x* exceeds about 0.2,^25,26^ is absent in the 2D *n* = 1 perovskite PEA_2_Pb(I_0.5_Br_0.5_)_4_.^17,18^ A kinetic explanation has been given of this improved photostability based on an increased halide migration barrier in PEA_2_Pb(I_0.5_Br_0.5_)_4_ in comparison to MAPb(I_0.5_Br_0.5_)_3_, by about 80 meV.^17^ Although such an increased energy barrier—about 3 times the room-temperature thermal energy of 25 meV—will definitely slow down halide segregation, it cannot explain the complete absence of such segregation. Instead, in this Letter, we will provide an equilibrium thermodynamic explanation for the increased photostability of PEA_2_Pb(I_0.5_Br_0.5_)_4_. To understand light-induced halide segregation in 3D mixed halide perovskites, we recently developed a unified thermodynamic theory that considers a free energy that is the sum of a compositional and a photocarrier free energy.^27^ In this theory, photocarriers can decrease their free energy by funneling into a low-band-gap phase that is nucleated out of a mixed parent phase. We applied the theory to a series of 3D mixed I–Br perovskites and could explain several experimental observations from the calculated composition–temperature (*x*–*T*) phase diagrams at different illumination intensities.^27^ For MAPb(I_1–*x*_Br_*x*_)_3_, we predicted a dependence of the threshold illumination intensity on composition and temperature and a temperature dependence of the threshold Br concentration for halide demixing that are qualitatively consistent with experimental results.^27^ In this Letter, we apply this thermodynamic theory to the 2D mixed halide perovskite PEA_2_Pb(I_1–*x*_Br_*x*_)_4_ and study its phase stability in the dark and under illumination. We start with a calculation of the Helmholtz compositional free energy and then construct the *x*–*T* phase diagrams of PEA_2_Pb(I_1–*x*_Br_*x*_)_4_ in the dark. After adding a photocarrier contribution, we construct the phase diagrams for different illumination intensities. Similar to MAPb(I_1–*x*_Br_*x*_)_3_, we predict the existence of an illumination intensity and Br concentration threshold for halide demixing. We find that, both in the dark and under illumination, PEA_2_Pb(I_1–*x*_Br_*x*_)_4_ is thermodynamically much more stable than MAPb(I_1–*x*_Br_*x*_)_3_, and we discuss the reasons for this enhanced stability.

To obtain the Helmholtz compositional mixing free energy of PEA_2_Pb(I_1–*x*_Br_*x*_)_4_, we first calculate within density functional theory (DFT) the mixing enthalpies Δ*U* per formula unit (f.u.) of all possible I–Br configurations at different Br concentrations *x* = 0, 1/8, 1/4, 3/8, 1/2, 5/8, 3/4, 7/8, and 1, according to eq 1 in the “Methods” section. The results are shown by circles in Figure 1a. We clearly identify an enthalpically preferred I–Br distribution where the Br anions are located at the equatorial sites of the Pb–X octahedral layers and the I anions at the axial sites. This leads to a situation where the most stable (unstable) configuration with the lowest (highest) enthalpy for each Br concentration *x* has the maximum number of Br (I) anions at equatorial sites. The configurations with the highest and lowest enthalpy for *x* = 0.5 are displayed in Figure 1b, where the top configuration is the most unstable (enthalpy given by the pink filled circle in Figure 1a), with all Br anions in the axial layer, and the bottom configuration is the most stable (enthalpy given by the green filled circle), with all Br anions in the equatorial layer.

**Figure 1:**
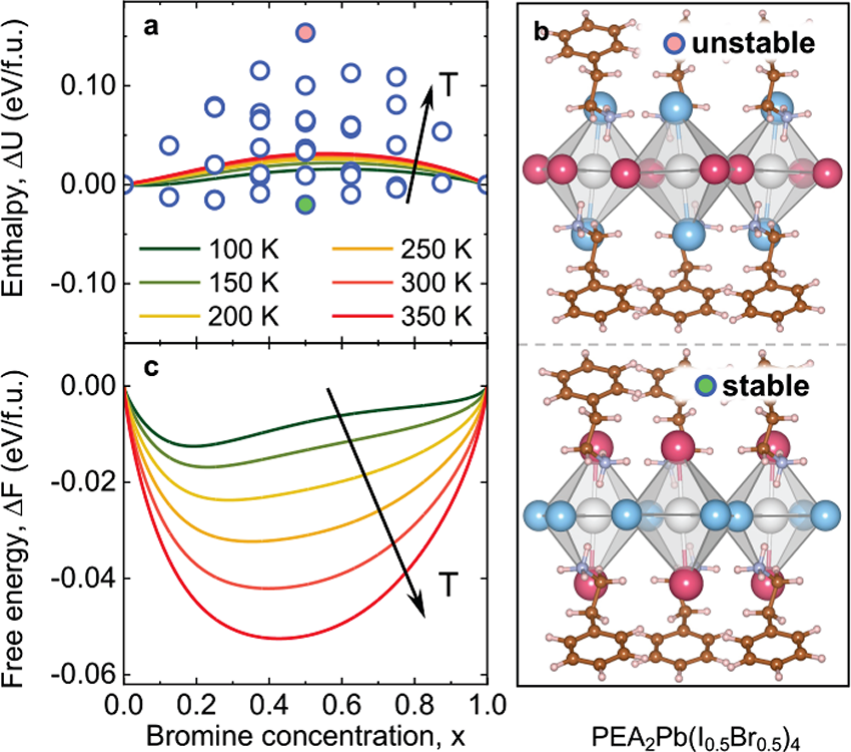
(a) Mixing enthalpy per formula unit (f.u.) as a function of Br concentration *x* for the 2D perovskite PEA_2_Pb(I_1–*x*_Br_*x*_)_4_. Circles: values calculated for each possible configuration of I and Br anions. Filled circles: values for the most unstable (pink) and stable (green) configurations for *x* = 0.5. Curves: results when using the quasichemical approximation (QCA) at different temperatures. (b) Atomic structures for the most unstable and stable I–Br configurations at *x* = 0.5. (c) Mixing free energy per f.u. at different temperatures as a function of Br concentration.

The preferential I–Br distribution can be explained by a volume effect. Due to the inequivalence of equatorial and axial sites, the substitution of smaller but more electronegative Br anions for I anions at equatorial and axial sites of the unit cell of PEA_2_PbI_4_ to form shorter Pb–Br bonds gives rise to different degrees of volume contraction (see Section S1 in the Supporting Information). For a given Br concentration, the volume of the unit cell tends to be smaller when the Br anions are placed at equatorial sites than when they are placed at axial sites. This is mainly due to the fact that each equatorial anion forms chemical bonds with two adjacent Pb cations, whereas each axial anion on one side forms a bond with an equatorial Pb cation, and on the other side, it is only weakly bonded to the organic layer. Substitution at equatorial sites will therefore lead to larger structural changes than substitution at axial sites. A reduced volume leads to an enhanced chemical bonding between the halide anions with surrounding cations, which results in a lower enthalpy.

By applying the quasichemical approximation (QCA)^27−29^ of binary alloying theory to the mixing enthalpies calculated at discrete *x*, we obtain the mixing enthalpy Δ*U*(*x*, *T*) as a continuous function of Br concentration *x* for different temperatures (lines in Figure 1a). Taking additionally the mixing entropy Δ*S*(*x*, *T*) into account, according to eq 2 in “Methods”, yields the compositional mixing free energy Δ*F*(*x*, *T*) per f.u. at different temperatures, displayed in Figure 1c. As a reference, the mixing enthalpy and free energy of 3D MAPb(I_1–*x*_Br_*x*_)_3_ are reproduced in Section S2 in the Supporting Information.

The composition–temperature, *x*–*T*, phase diagram of a mixed halide perovskite in the dark can, analogously to ordinary binary mixtures, be constructed by collecting the points of common tangent (binodal) and the inflection points (spinodal) of the compositional mixing free energy in *x*–*T* space.^27,29^ In Figure 2a,b, we show the phase diagrams in the dark of 3D MAPb(I_1–*x*_Br_*x*_)_3_ (reproduced from ref (27)) and 2D PEA_2_Pb(I_1–*x*_Br_*x*_)_4_, respectively. The blue curve in the phase diagrams is the binodal, separating the metastable region (gray) from the stable region (white). The red curve is the spinodal, separating the unstable region (pink) from the metastable region. The position where the binodal and spinodal meet is the critical point (*x*_*c*_, *T*_*c*_). The predicted critical temperature *T*_*c*_ of 2D PEA_2_Pb(I_1–*x*_Br_*x*_)_4_ is about 161 K, which is much lower than that of 3D MAPb(I_1–*x*_Br_*x*_)_3_ (266 K).^27^ This shows that PEA_2_Pb(I_1–*x*_Br_*x*_)_4_ is thermodynamically much more stable in the dark. The superior phase stability of PEA_2_Pb(I_1–*x*_Br_*x*_)_4_ is explained by the favorable I–Br distribution, as discussed above and shown in Figure 1. Since in this favorable distribution the I and Br anions are already well demixed (the I anions prefer to be at axial sites and the Br anions prefer to be at equatorial sites), the enthalpic driving force for a further demixing is strongly reduced.

**Figure 2:**
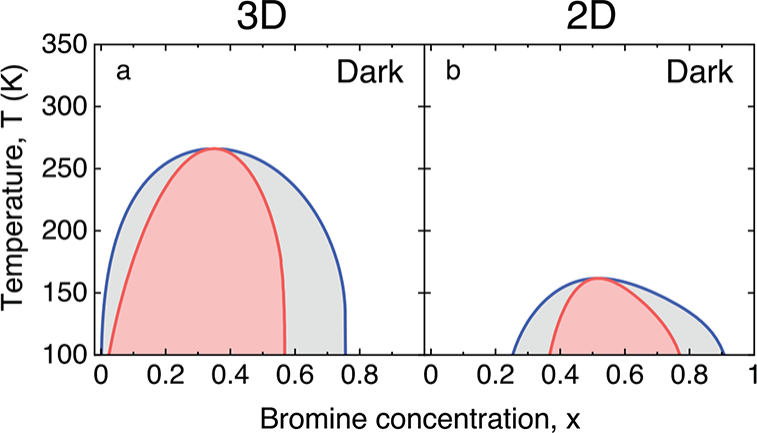
Phase diagrams for halide segregation in the dark in the temperature window *T* = 100–150 K of (a) the 3D perovskite MAPb(I_1–*x*_Br_*x*_)_3_ and (b) the 2D perovskite PEA_2_Pb(I_1–*x*_Br_*x*_)_4_. Blue curves: binodals separating the metastable (gray) and stable (white) regions. Red curves: spinodals separating the unstable (pink) and metastable regions.

The presence of photocarriers under illumination requires addition of a photocarrier free energy contribution to the compositional mixing free energy.^27^ Assuming segregation into two phases with different Br concentrations *x*_1_ and *x*_2_, this free energy contribution is equal to the sum over the two phases of the number of photocarriers in each phase multiplied by the band gap of the phase; see eq 3 in “Methods”. The band gaps of PEA_2_Pb(I_1–*x*_Br_*x*_)_4_ and MAPb(I_1–*x*_Br_*x*_)_3_ are taken from experiment (see Section S3 in the Supporting Information). The band gap of PEA_2_Pb(I_1–*x*_Br_*x*_)_4_ can be well described by the function *E*_*g*_(*x*) = 2.37(1 – *x*) + 3.03*x* – 0.33*x*(1 – *x*) eV,^21^ which interpolates between the band gap of 2.37 eV for PEA_2_PbI_4_ and 3.03 eV for PEA_2_PbBr_4_. The band gap of MAPb(I_1–*x*_Br_*x*_)_3_ is smaller and described by the function *E*_*g*_(*x*) = 1.57(1 – *x*) + 2.29*x* – 0.33*x*(1 – *x*) eV,^30^ with band gaps of 1.57 eV for MAPbI_3_ and 2.29 eV for MAPbBr_3_. The dependence of the band gap on the Br concentration *x* in PEA_2_Pb(I_1–*x*_Br_*x*_)_4_ is slightly smaller than in MAPb(I_1–*x*_Br_*x*_)_3_, but we will see that this small difference has an important effect on the phase diagrams under illumination.

The photocarrier densities in the two phases are obtained from a thermally governed band-gap-dependent redistribution over the two phases and a balance between the photocarrier generation and annihilation processes in the two phases; see eqs 4 and 5 in “Methods”. We make the simplifying assumption that the photocarrier generation rate *G* is the same in the two phases and is for a thin film given by *G* = *IαV*/*hν*, where *I* is the illumination intensity (*I* ≈ 100 mW cm^–2^ for 1 Sun), α is the absorption coefficient, *V* is the volume per f.u., and *hν* is the photon energy. The annihilation of photocarriers is characterized by monomolecular and bimolecular recombination in the two phases, where the rate constants, given by the inverse photocarrier lifetime 1/τ and *k*, respectively, are assumed to be phase-independent. For both MAPb(I_1–*x*_Br_*x*_)_3_ and PEA_2_Pb(I_1–*x*_Br_*x*_)_4_, we take the values α = 10^–5^ cm^–1^, *hν* = 3 eV,^26,31^*k* = 10^–10^ cm^3^ s^–1^,^31,32^ and *V* = 2.5 × 10^–22^ cm^3^,^27^ applicable for standard MAPbI_3_ and PEA_2_PbI_4_ films. Since the photocarriers in PEA_2_Pb(I_1–*x*_Br_*x*_)_4_ are confined in the quantum-well-like inorganic layer, we take an effective volume *V* for PEA_2_Pb(I_1–*x*_Br_*x*_)_4_ per f.u. that is approximately equal to the volume of MAPbI_3_ per f.u. For the photocarrier lifetime, we take the experimental values τ = 100 ns^32^ and 1 ns^31,33^ for MAPb(I_1–*x*_Br_*x*_)_3_ and PEA_2_Pb(I_1–*x*_Br_*x*_)_4_, respectively. The strong decrease in photocarrier lifetime when going from a 3D to a 2D perovskite might be ascribed to excitonic effects^31^ or a low crystal quality with deep traps. To disentangle the effects on the photostability of the different dependencies of the band gap on Br concentration and the different photocarrier lifetimes, we first use photocarrier lifetimes of 100 ns for both MAPb(I_1–*x*_Br_*x*_)_3_ and PEA_2_Pb(I_1–*x*_Br_*x*_)_4_ and next take the appropriate lifetime of 1 ns for PEA_2_Pb(I_1–*x*_Br_*x*_)_4_.

The halide segregation in mixed I–Br perovskites under illumination is a consequence of a decreased free energy by accumulation of photocarriers in a low-band-gap nucleated I-rich phase,^26,27^ where the driving force for halide demixing is the band gap difference between the parent mixed phase and the nucleated low-band-gap I-rich phase.^26^ The band gap differences for different halide compositions in MAPb(I_1–*x*_Br_*x*_)_3_ are mainly caused by changes in the energy of the valence band maximum, which increases with increasing I concentration.^26^ In PEA_2_Pb(I_1–*x*_Br_*x*_)_4_, the energy of the valence band maximum increases and the energy of the conduction band minimum decreases with increasing I concentration.^34^ We thus conclude that in MAPb(I_1–*x*_Br_*x*_)_3_ it will be mainly the photogenerated holes, while in PEA_2_Pb(I_1–*x*_Br_*x*_)_4_, it will be both the photogenerated electrons and holes that can reduce their free energy by funneling into I-rich domains. In the presence of illumination, the usual method of finding binodals and spinodals—used to obtain the phase diagram in the dark of Figure 2—is not applicable, and instead, a more sophisticated procedure should be used to find the minima of the total (compositional plus photocarrier) free energy under the constraints ϕ_1_ + ϕ_2_ = 1 (ϕ_1_ and ϕ_2_ are the volume fractions of the two phases) and ϕ_1_*x*_1_ + ϕ_2_*x*_2_ = *x*.^27^

Figure 3a–f shows the composition–temperature phase diagrams for PEA_2_Pb(I_1–*x*_Br_*x*_)_4_ and MAPb(I_1–*x*_Br_*x*_)_3_ at increasing illumination intensities *I* = 0.1, 1, and 10 Sun, taking τ = 100 ns for both perovskites. In comparison to the phase diagrams in the dark (see Figure 2), the spinodals in both perovskites only slightly change by the illumination. By contrast, the binodals change considerably. As already found in our previous analysis,^27^ for 3D MAPb(I_1–*x*_Br_*x*_)_3_, two types of binodals are obtained, a compositional binodal (blue curve) and a light-induced binodal (green curve). When the compositional binodal is crossed by increasing *x* or decreasing *T*, nucleation of a phase that is more Br-rich than the parent phase becomes favorable, as indicated by the dashed blue line. When the light-induced binodal is crossed by increasing *x* or decreasing *T*, a nearly I-pure phase is nucleated, as indicated by the dashed green line. The location where the compositional and light-induced binodals meet was suggested to be a triple point where two phases with different halide compositions may be nucleated out of the parent phase,^27^ as indicated by the three differently colored dots. In 2D PEA_2_Pb(I_1–*x*_Br_*x*_)_4_, only the light-induced binodal exists under the investigated illumination intensities for a photocarrier lifetime of 100 ns.

**Figure 3:**
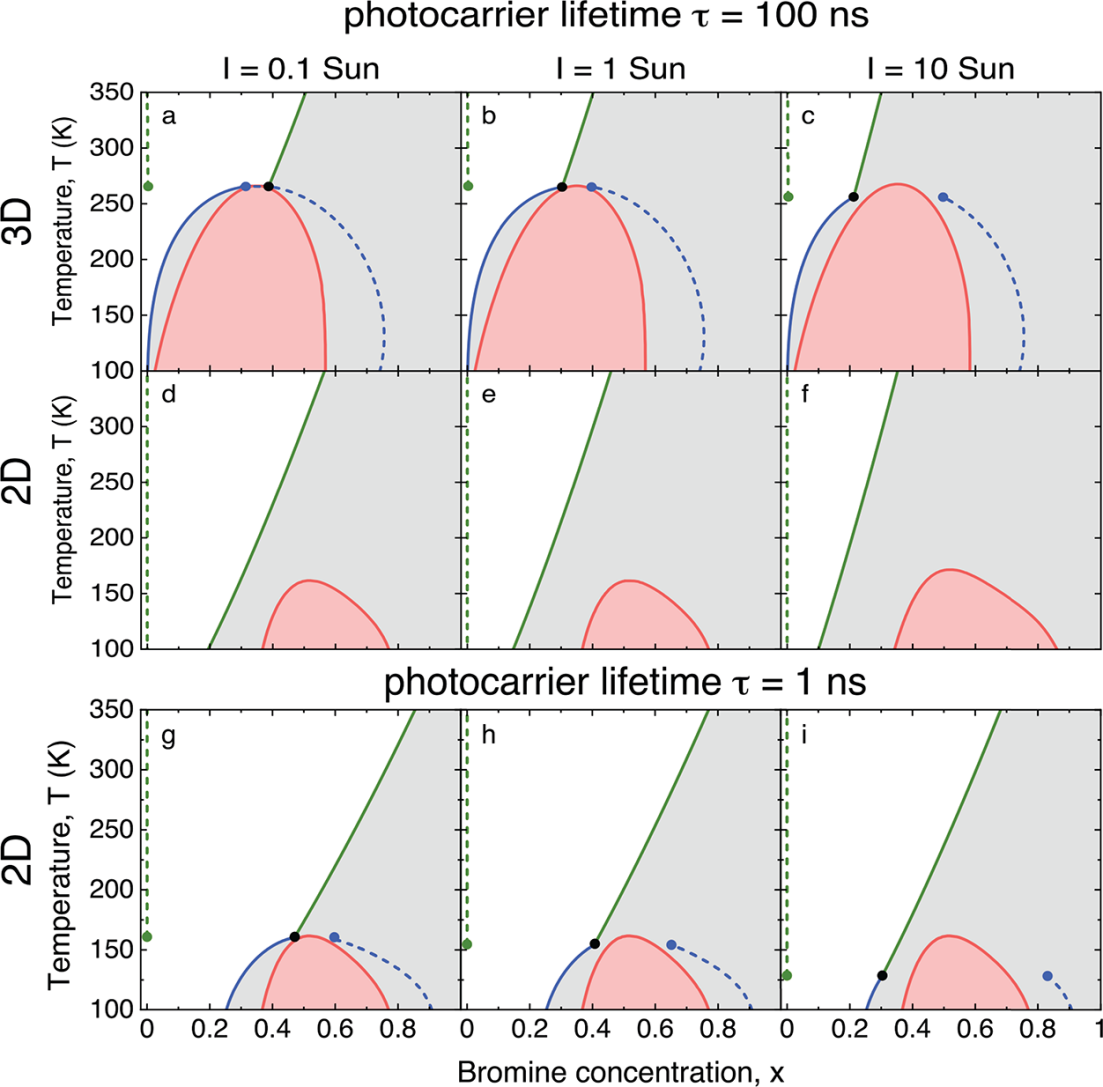
(a–f) Phase diagrams of 3D MAPb(I_1–*x*_Br_*x*_)_3_ and 2D PEA_2_Pb(I_1–*x*_Br_*x*_)_4_ perovskites for illumination intensities *I* = 0.1, 1, and 10 Sun, for a photocarrier lifetime τ = 100 ns in both perovskites. (g–i) Phase diagrams of PEA_2_Pb(I_1–*x*_Br_*x*_)_4_ at the same illumination intensities but for the appropriate photocarrier lifetime τ = 1 ns. Red curves: spinodals separating the metastable (gray) and unstable (pink) regions. Full blue and green curves: binodals separating the stable (white) and metastable regions. The blue (green) full curves indicate the compositional (light-induced) binodals. When the metastable region is entered by crossing the compositional (light-induced) binodals, a phase with a Br concentration indicated by the dashed blue (green) lines is nucleated. The dots indicate the possible coexistence of three phases: the parent phase (black dots) and two types of nucleated phases with different Br concentrations (blue and green dots).

By comparing Figure 3d–f to Figure 3a–c, we see that at 300 K (room temperature) the light-induced binodals of PEA_2_Pb(I_1–*x*_Br_*x*_)_4_ occur at a higher Br concentration *x* than those of MAPb(I_1–*x*_Br_*x*_)_3_. This means that PEA_2_Pb(I_1–*x*_Br_*x*_)_4_ is thermodynamically more stable than MAPb(I_1–*x*_Br_*x*_)_3_ for comparable illumination intensities and photocarrier lifetimes. This increased photostability is caused by the smaller band gap difference between the parent and nucleated phase in PEA_2_Pb(I_1–*x*_Br_*x*_)_4_ as compared to MAPb(I_1–*x*_Br_*x*_)_3_, which leads to a smaller driving force for halide demixing. However, this effect alone cannot explain the absence of halide segregation for *x* = 0.5 in PEA_2_Pb(I_1–*x*_Br_*x*_)_4_ at room temperature and under 1 Sun illumination,^17,18^ since Figure 3e shows that PEA_2_Pb(I_0.5_Br_0.5_)_4_ is metastable under these conditions (gray region), so that halide demixing is still expected to occur.

Figure 3g–i shows for PEA_2_Pb(I_1–*x*_Br_*x*_)_4_ results comparable to Figure 3d–f, but for the appropriate experimentally determined photocarrier lifetime τ = 1 ns.^31,33^ We observe that the decrease in lifetime from 100 to 1 ns has almost no effect on the spinodals but leads to a strong shift of the binodals to higher Br concentration *x*. Like in 3D MAPb(I_1–*x*_Br_*x*_)_3_, a compositional binodal and a triple point appear. The increased photostability by the reduction of the photocarrier lifetime is caused by the reduced concentration of photocarriers and the concurring reduced driving force for phase segregation. The reduction in lifetime by a factor 100 roughly corresponds to a reduction of the illumination intensity by the same factor, as illustrated by the similarity of the phase diagrams of Figure 3d,i.

When the light-induced binodal is crossed, a metastable region is entered, leading to a threshold for halide demixing in *x*–*T*–*I* phase space, beyond which demixing is expected.^27^ The threshold illumination intensity in PEA_2_Pb(I_1–*x*_Br_*x*_)_4_ for *x* = 0.5, *T* = 300 K, and τ = 1 ns is calculated to be about *I* = 90 Sun, substantially higher than in MAPb(I_1–*x*_Br_*x*_)_3_ (about 0.02 Sun).^27^ The threshold Br concentration *x* in PEA_2_Pb(I_1–*x*_Br_*x*_)_4_ at 300 K under 1 Sun illumination is about 0.7, which is a factor of about 2 larger than in MAPb(I_1–*x*_Br_*x*_)_3_.^27^ Because *x* = 0.5 is below the threshold Br concentration for halide demixing, PEA_2_Pb(I_1–*x*_Br_*x*_)_4_ is predicted by our theory to be thermodynamically stable at 300 K under 1 Sun illumination, in accordance with the experimental observations.^17,18^ We note that this prediction is obtained within an equilibrium thermodynamic theory and should therefore be contrasted to the kinetic explanation of increased photostability based on an increased barrier for halide migration.^17^ Recently, it has been reported that the Dion-Jacobson 2D perovskite (PDMA)Pb(I_1–*x*_Br_*x*_)_4_ (PDMA: 1,4-phenylenedimethanammonium) demixes under illumination for *x* = 0.5.^35^ This might be ascribed to a higher photocarrier lifetime in (PDMA)Pb(I_1–*x*_Br_*x*_)_4_ in comparison to PEA_2_Pb(I_1–*x*_Br_*x*_)_4_, possibly because of a lower concentration of defects. The bivalent nature of the PDMA cation might result in a better crystallinity of (PDMA)Pb(I_1–*x*_Br_*x*_)_4_ as compared to PEA_2_Pb(I_1–*x*_Br_*x*_)_4_, where PEA is monovalent. To resolve this issue, it would be helpful if the photocarrier lifetime in (PDMA)Pb(I_1–*x*_Br_*x*_)_4_ is determined.

In summary, using a unified thermodynamic theory, we have proposed an equilibrium thermodynamic origin of the superior phase stability of the 2D mixed halide perovskite PEA_2_Pb(I_1–*x*_Br_*x*_)_4_ as compared to its 3D counterpart perovskite MAPb(I_1–*x*_Br_*x*_)_3_. We have found that PEA_2_Pb(I_1–*x*_Br_*x*_)_4_ is thermodynamically more stable than MAPb(I_1–*x*_Br_*x*_)_3_, both in the dark and under illumination. Several factors can explain this difference. The improved phase stability of PEA_2_Pb(I_1–*x*_Br_*x*_)_4_ in the dark can be explained by an energetically favorable I–Br distribution, where I and Br anions are preferably located on different types of lattice sites. The smaller band gap difference of the mixed parent phase and the nucleated low-band-gap phase, and particularly a much shorter photocarrier lifetime, are identified as the reasons for the enhanced phase stability of PEA_2_Pb(I_1–*x*_Br_*x*_)_4_ under illumination. These findings provide important fundamental insight into the suppressed halide segregation in PEA_2_Pb(I_1–*x*_Br_*x*_)_4_. Such insight is critical in the quest for long-term stable mixed halide perovskites for use in optoelectronic devices.

## Methods

### Calculation of Total Energies

To calculate the total energies of PEA_2_Pb(I_1–*x*_Br_*x*_)_4_, we start from a periodic unit cell of PEA_2_PbI_4_ containing four formula units, with two parallel inorganic Pb–I octahedral layers in the equatorial plane and two organic PEA bilayers intercalating along the axial direction.^7^ We then replace I anions by Br anions at different Br concentrations *x* = 0, 1/8, 1/4, 3/8, 1/2, 5/8, 3/4, 7/8, 1. For each possible configuration, we take the same halide distribution for the two parallel inorganic layers. The total number of possible configurations then becomes 2^8^ = 256. With a perfect *D*_4*h*_ symmetry for each inorganic layer, the total number of inequivalent configurations is reduced to 45. The small deviation from *D*_4*h*_ symmetry due to the presence of the spacer PEA cations is not important because the PEA cation is not incorporated into the inorganic layer, unlike in the 3D case. We note that for each configuration, the whole structure is optimized without symmetry restrictions.

The total energy calculations are performed within density functional theory (DFT), using the projector augmented wave (PAW)^36^ method as implemented in the Vienna Ab initio Simulation Package (VASP).^37^ The used electronic exchange-correlation interaction is described by the Perdew–Burke–Ernzerhof (PBE) functional within the generalized gradient approximation (GGA).^38^ We use 6 × 6 × 2 *k*-point Brillouin zone samplings and a plane-wave kinetic energy cutoff of 500 eV. The D3 correction^39^ is used to describe the van der Waals interactions between the organic PEA bilayers and the inorganic layers. The shape, volume, and atomic positions of each possible configuration are fully optimized. The structure files of the optimized configurations can be found in the Supporting Information. Energy and force convergence criteria of 0.01 meV and 0.01 eV/Å, respectively, are used in all calculations.

### Calculation of the Mixing Free Energy in the Dark

The mixing enthalpy per f.u. Δ*U*_*j*_ of inequivalent I–Br configurations *j* = 1, 2, ..., *J* for 2D PEA_2_Pb(I_1–*x*_Br_*x*_)_4_ is calculated by
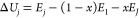
1where *E*_*j*_, *E*_1_, and *E*_*J*_ are the total energies per f.u. of the inequivalent mixed I–Br, the pure I, and the pure Br configurations, respectively. The results are displayed by circles in Figure 1a. We apply the quasichemical approximation (QCA)^28^ to obtain the Helmholtz compositional free energy Δ*F*(*x*, *T*) as functions of the Br concentration *x* and temperature *T*,
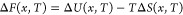
2where Δ*U*(*x*, *T*) and Δ*S*(*x*, *T*) are the mixing enthalpy and entropy, respectively. Further computational details can be found in ref (27).

### Calculation of the Free Energy under Illumination

Like in ref (27), we minimize the total free energy per f.u. under illumination, which consists of the sum over the two phases of a compositional and a photocarrier free energy:
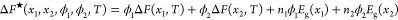
3Here, ϕ_1_ and ϕ_2_ are the volume fractions of the two phases, and *x*_1_ and *x*_2_ are the Br concentrations of the two phases, which have different band gaps *E*_*g*_(*x*_1_) and *E*_*g*_(*x*_2_). Neglecting the small volume difference per f.u. between the two phases yields the conditions ϕ_1_ + ϕ_2_ = 1 and ϕ_1_*x*_1_ + ϕ_2_*x*_2_ = *x*. Depending on the band gaps of the two phases, the photocarriers thermally redistribute over the two phases with different densities (numbers of photocarriers per f.u.) *n*_1_ and *n*_2_. Demanding local charge neutrality, the photocarrier densities correspond in each phase to the densities of photogenerated electrons as well as holes. Since *n*_1_, *n*_2_ ≪ 1, we can use Boltzmann statistics:
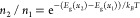
4where *k*_*B*_*T* is the thermal energy. We assume that the diffusion length of photocarriers is larger than the feature size of domains so that we can take the distribution of photocarriers in each phase to be uniform. In equilibrium, the total generation rates of photocarriers are equal to the total annihilation rates by monomolecular and bimolecular recombination in the two phases:
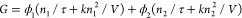
5

The techniques used in finding the—local and global—minima of the total free energy equation (eq 3), needed to obtain the binodals and spinodals in Figures 2 and 3, and the thresholds for halide demixing are the same as in ref (27) and are described there in detail.

## Supporting Information Available

Crystal volumes calculated by DFT; compositional mixing enthalpy and free energy of MAPb(I_1–*x*_Br_*x*_)_3_; band gaps of PEA_2_Pb(I_1–*x*_Br_*x*_)_4_ and MAPb(I_1–*x*_Br_*x*_)_3_, including Figures S1–S3(PDF)
Structure files of PEA_2_Pb(I_1–*x*_Br_*x*_)_4_ (ZIP)
