# Supplementary material for: Thermodynamic Origin of the Photostability of the Two-Dimensional Perovskite PEA2Pb(I1–xBrx)4
Source: ACS Energy Lett. 2023 Jan 13;8(2):943–9. doi: 10.1021/acsenergylett.2c02463 (PMC9926482; doi:10.1021/acsenergylett.2c02463)
Supplement: Supplementary file 1 — nz2c02463_si_001.pdf [file nz2c02463_si_001.pdf]

Supporting Information: Thermodynamic origin of the photostability of the two-dimensional perovskite $PEA_2Pb(I_{1-x}Br_x)_4$

Zehua Chen,[†,‡] Haibo Xue,[†,‡] Geert Brocks,[†,‡,¶] Peter A. Bobbert,[*,‡,§] and Shuxia Tao[*,†,‡]

†*Materials Simulation and Modelling, Department of Applied Physics, Eindhoven University of Technology, 5600 MB Eindhoven, The Netherlands*

‡*Center for Computational Energy Research, Department of Applied Physics, Eindhoven University of Technology, P.O. Box 513, 5600 MB Eindhoven, The Netherlands*

¶*Computational Materials Science, Faculty of Science and Technology and MESA+ Institute for Nanotechnology, University of Twente, P.O. Box 217, 7500 AE Enschede, The Netherlands*

§*Molecular Materials and Nanosystems, Eindhoven University of Technology, P.O. Box 513, NL-5600 MB Eindhoven, The Netherlands*

E-mail: P.A.Bobbert@tue.nl; S.X.Tao@tue.nl

S1. Crystal volumes calculated by DFT

Figure S1 shows the calculated volumes per formula unit (f.u.) at each discrete Br concentration x = 0, 1/8, 1/4, 3/8, 1/2, 5/8, 3/4, 7/8, 1, for the most stable and unstable configurations of $PEA_2Pb(I_{1-x}Br_x)_4$. The most unstable and most stable configurations for $x = 0.5$ are displayed in Figure 1b in the main text. The replacement of all the equatorial I anions of PEA_2PbI_4 by smaller Br anions creates the most stable configuration for $x = 0.5$. The lattice constant in the equatorial plane is then reduced by $\sim 3\%$ due to the increased electrostatic potential energy arising from the higher electronegativity of Br anions as compared to I anions. As expected, the lattice in the axial direction remains approximately unchanged, with a $\sim 0.25\%$ compression. This leads to a decrease of volume by $\sim 6\%$ as compared to the pure I configuration. By contrast, when all the axial I anions of PEA_2PbI_4 are replaced by smaller Br anions, the most unstable configuration for $x = 0.5$ is created. Both in the equatorial plane and in the axial direction the lattice constant remains almost unchanged, with $\sim 0.5\%$ compression. As a result, the cell volume is compressed only by $\sim 1.5\%$ in comparison to the pure I configuration. The difference in the degree of volume contraction indicates that the volume changes are more sensitive to the equatorial anions than to the axial anions. This also holds for other compositions.

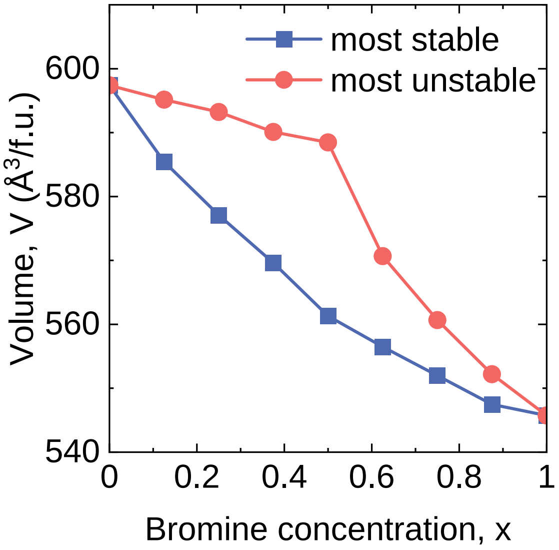

Figure S1: Calculated volumes per formula unit (f.u.) of $PEA_2Pb(I_{1-x}Br_x)_4$ for the most stable (blue) and unstable (red) configurations as a function of x. The most stable (unstable) configurations for each discrete Br concentration at $x = 0$, 1/8, 1/4, 3/8, 1/2, 5/8, 3/4, 7/8, and 1 are those with the maximum number of Br (I) anions present at the equatorial (axial) sites of the inorganic layer.

S2. Compositional mixing enthalpy and free energy of $MAPb(I_{1-x}Br_x)_3$

In Figure S2 we reproduce the compositional mixing enthalpy $\Delta U(x, T)$ and free energy $\Delta F(x, T)$ calculated in Ref.[1] , which enters Equation (3). This mixing free energy was calculated following the procedure outlined in Ref.[2] , using density functional theory (DFT) with a thermodynamic analysis performed within the generalized quasichemical approximation (QCA).[3]

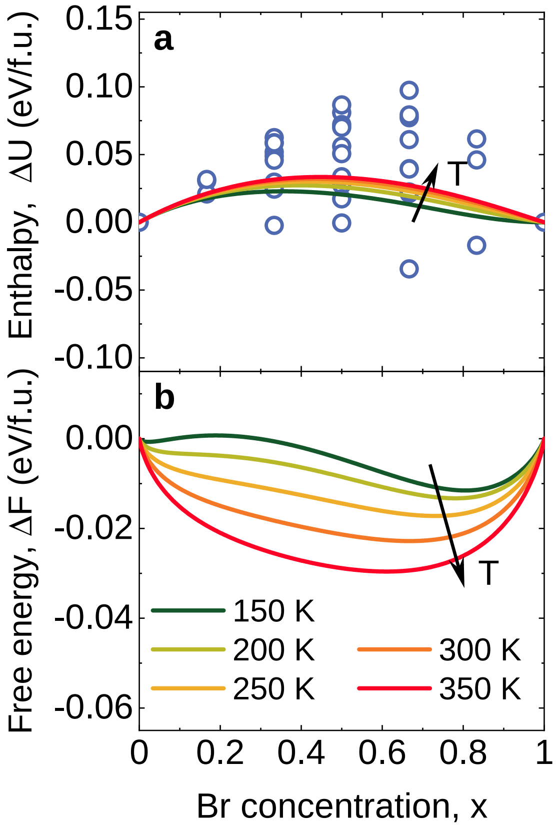

Figure S2: **a** Mixing enthalpy per formula unit (f.u.) of $MAPb(I_{1-x}Br_x)_3$ as a function of Br concentration x. Circles: values calculated for each possible configuration. Curves: results for the QCA at different temperatures. **b** Mixing free energy per f.u. as a function of Br concentration at different temperatures.

S3. Band gaps

In Figure S3 we display the experimentally determined band gaps as a function of Br concentration x for $PEA_2Pb(I_{1-x}Br_x)_4$[4] and $MAPb(I_{1-x}Br_x)_3$.[5] The formulas of the band gaps for both compounds are accurately described by

$$E_g(x) = (1-x)E_g(x=0) + xE_g(x=1) - bx(1-x), \tag{S1}$$

where $E_g(x=0)$ and $E_g(x=1)$ are the band gaps of the pure I and pure Br perovskites, respectively, and b is the bowing parameter.

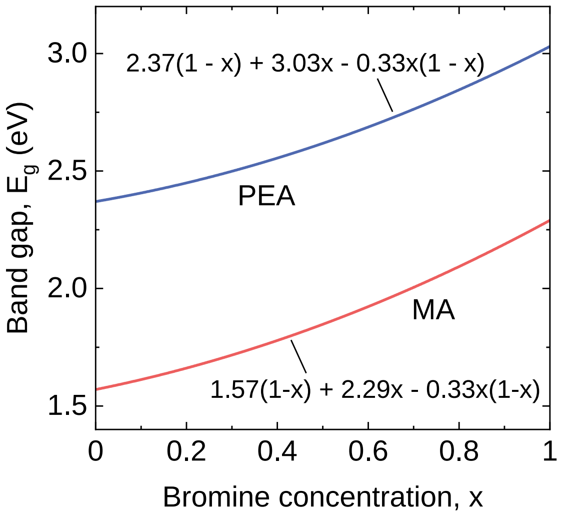

Figure S3: Band gap as a function of Br concentration x for $PEA_2Pb(I_{1-x}Br_x)_4$ and $MAPb(I_{1-x}Br_x)_3$.

References

(1) Chen, Z.; Brocks, G.; Tao, S.; Bobbert, P. A. Unified theory for light-induced halide segregation in mixed halide perovskites. *Nature communications* **2021**, *12*, 1–10.

(2) Brivio, F.; Caetano, C.; Walsh, A. Thermodynamic origin of photoinstability in the $CH_3NH_3Pb(I_{1-x}Br_x)_3$ hybrid halide perovskite alloy. *The journal of physical chemistry letters* **2016**, *7*, 1083–1087.

(3) Sher, A.; van Schilfgaarde, M.; Chen, A.-B.; Chen, W. Quasichemical approximation in binary alloys. *Physical Review B* **1987**, *36*, 4279.

(4) Zhang, S.; Lin, Z.-Y.; Shi, E.; Finkenauer, B. P.; Gao, Y.; Pistone, A. J.; Ma, K.; Savoie, B. M.; Dou, L. Quantifying anionic diffusion in 2D halide perovskite lateral heterostructures. *Advanced Materials* **2021**, *33*, 2105183.

(5) Noh, J. H.; Im, S. H.; Heo, J. H.; Mandal, T. N.; Seok, S. I. Chemical management for colorful, efficient, and stable inorganic–organic hybrid nanostructured solar cells. *Nano letters* **2013**, *13*, 1764–1769.
